# Losartan alters osteoblast differentiation and increases bone mass through inhibition of TGF*B* signalling in vitro and in an OIM mouse model

**DOI:** 10.1016/j.bonr.2024.101795

**Published:** 2024-07-25

**Authors:** Mai Morita, Fawaz Arshad, Lewis A. Quayle, Christopher N. George, Diane V. Lefley, Ivo Kalajzic, Meena Balsubramanian, Tugba Cebe, Gwen Reilly, Nicolas J. Bishop, Penelope D. Ottewell

**Affiliations:** a Mellanby Centre for Musculoskeletal Research and Division of Clinical Medicine, University of Sheffield, Beech Hill Road, Sheffield, UK; b Department of Computing, Sheffield Hallam University, Cantor Building, Arundel Street, Sheffield, UK; c Reconstructive Sciences, UConn Health, Farmington, CT. USA; d Highly specialised Osteogenesis Imparfecta Service, Sheffield Children's NHS Foundation Trust, Sheffield, UK; e INSIGNIO Institute for in silico Medicine and the Kroto Research Institute, Department of Materials Science and Engineering, University of Sheffield, Sheffield, UK

**Keywords:** Osteogenesis Imperfecta, Losartan, TGFβ, Bone

## Abstract

Excessive production of Transforming Growth Factor β (TGFβ) is commonly associated with dominant and recessive forms of OI. Previous reports have indicated that administration of TGFβ-targeted antibodies maybe of potential therapeutic benefit to OI patients. However, direct targeting of TGFβ is likely to cause multiple adverse effects including simulation of autoimmunity. In the current study we use patient-derived normal and OI fibroblasts, osteoblasts and OIM mouse models to determine the effects of Losartan, an angiotensin II receptor type 1 (AT1) antagonist, on TGFβ signalling and bone morphology in OI. In OIM mice bred on a mixed background administration of 0.6 g/L losartan for 4 weeks was associated with a significant reduction in TGFβ from 79.2 g/L in the control to 60.0 ng/ml following losartan (*p* < 0.05), reduced osteoclast activity as measured by CTX from 275.9 ng/ml in the control to 157.2 ng/ml following 0.6 g/L of losartan (p < 0.05) and increased cortical bone thickness (*P* < 0.001). Furthermore in OIM mice bred on a C57BL/6 background 0.6 g/L losartan increased trabecular bone volume in the tibiae (*P* < 0.05) and the vertebrae (*P* < 0.01), increased cortical bone thickness (*P* < 0.001) reduced the trabecular pattern factor (*P* < 0.01 and P < 0.001 for the tibiae and vertebrae respectively), reduced osteoclast (*P* < 0.05) and osteoblast (P < 0.01) numbers as well as reducing the area of bone covered by these cell types. Interestingly, losartan did not affect immune cells infiltrating into bone, nor did this drug alter TGFβ signalling in normal or OI fibroblasts. Instead, losartan reduced SMAD2 phosphorylation in osteoblasts, inhibiting their ability to differentiate. Our data suggest that losartan may be an effective treatment for the bone-associated dysmorphia displayed in OI whilst minimising potential adverse immune cell-related effects.

## Highlights

• Losartan reverses excessive serum concentrations of TGFβ observed in OI.
• Losartan reduces osteoclast/osteoblast numbers and activity as well as osteoblast differentiation without adversely effecting immunity.
• Low dose losartan increases trabecular bone density and cortical thickness reducing tibial fractures in OIM mice.
• Our data suggest that losartan may be a suitable candidate for repurposing as a new treatment for OI.

## 1 Introduction

Osteogenesis Imperfecta (OI) is the commonest inherited cause of bone fragility with an incidence of approximately 1/15,000–1/20,000 (Bregou Bourgeois et al., 2016). People with OI suffer bone fragility due largely to low bone mass and altered bone microarchitecture, increased bone material brittleness and increased bone turnover. This leads to fractures, pain and deformity; sarcopenia causing fatigue and poor endurance; aortic root dilatation and hearing loss. The range of clinical phenotype is broad with severely affected individuals at risk of early death e.g. from respiratory failure in infancy, or progressively deforming bone disease that leaves them with significantly reduced height, permanently wheelchair bound with scoliosis, basilar invagination and intractable pain. Even the more mildly affected individuals have an increased risk of fracture and suffer with easy fatigability. Some 85–90 % of cases are caused by pathological variants in the type I collagen genes, but at least 20 genetic origins have now been identified (Garibaldi et al., 2022).

OI patients have limited treatment options and traditionally these have been focused on increasing bone mass with the aim of reducing fracture risk. The most widely used pharmacological agents are bisphosphonates and multiple studies have shown that this class of agents are effective at increasing bone mass. However, their anti-fracture efficacy is less clear with two studies of mildly affected children demonstrating reduced fracture rates but little/no evidence for anti-fracture benefit in adults or more severely affected children (Hald et al., 2015; Marom et al., 2020; Dwan et al., 2014). Denosumab, a RANKL targeted antibody that prevents maturation of osteoblasts, halting bone resorption, and has a licence for use in post-menopausal osteoporosis (Reid and Billington, 2022) has been considered a strong drug candidate for OI patients. This drug may be especially useful for patients with the bisphosphonate unresponsive OI-VI subtype. Evidence suggests that denosumab effectively improves bone mineral density but not fracture rates in patients with OI. Although, some studies report hypercalciuria and moderate hypercalcemia as non-severe adverse effects (Majdoub et al., 2023) a pharma-sponsored phase II/III trial of denosumab in children with OI was terminated in September 2021 after episodes of severe hypercalcaemia in a number of children were reported. Treatment with denosumab is also complicated as removal of this drug leads to a rebound effect where patients experience an increase in bone resorption (Majdoub et al., 2023). Although it has been suggested that bisphosphonates can be used to prevent this bone rebound effect neither bisphosphonates or denosumab appear to provide anti-fracture benefit.

Taking the opposite approach of building bone through activating osteoblastigenesis with parathyroid hormone has been investigated in a placebo-controlled trial in adults with OI (Orwoll et al., 2014). This trial demonstrated an increase in lumber spine areal bone mineral density (LSaBMD) in treated as opposed to untreated patients, but there was no difference in self-reported fracture rates. Furthermore, in a study designed to both increase bone formation as well as inhibit bone resorption using the anti-sclerostin antibody, Setrusumab, preliminary data published from a phase II study in adults showed increased LSaBMD (Glorieux et al., 2017). These promising results have led to larger phase III studies in adults and children, however these studies are yet to formally report and no data on fracture are available. Therefore, to date, there is a lack of effective treatments for OI patients.

Excessive TGFβ signalling is becoming established as a common mechanism in OI (Grafe et al., 2014). Recent studies in murine OI models showed increased TGFβ-pathway signalling activity – cause unknown - and increased expression of TGFβ target genes (Grafe et al., 2014; Bianchi et al., 2015; Greene et al., 2021). Use of a murine pan-TGFβ neutralising antibody 1D11 in a severe OI mouse model (Crtap−/−) reduced bone resorption (CTX biomarker 25 % ± 5 % lower) and significantly increased spine bone volume (235 % increase in BV/TV) (Song et al., 2022). The same antibody also corrected high bone turnover and improved biomechanical properties in a COL1A2^tm1.Mcbr^ OI mouse model (Greene et al., 2021). Similar results were found in human studies, with excessive TGFβ signalling identified as a top dysregulatory event in bone and a major pathogenic mechanism in children with type III OI. In a phase 1 clinical trial, administration of the human anti-TGFβ antibody fresolimumab at a single dose of either 1 mg or 4 mg to eight adults with moderate or severe OI was well tolerated but had variable effects on bone mass according to OI type, with type IV (moderate OI) patients showing increased LSaBMD; those with type III and VIII (severe OI) did not show such changes (Song et al., 2022). It therefore appears that excessive TGFβ plays a significant role in OI pathophysiology and there may be potential for using TGFβ inhibitors to treat OI patients.

Losartan is an angiotensin II Type 1 receptor antagonist used to treat hypertension, cardiac failure and renal failure with proteinuria. Both human and animal studies have shown that administration of losartan in settings where TGFβ is elevated can result in TGFβ reduction with consequent functional effects: Inhibition of the TGFβ signalling pathway in preclinical models where TGFβ signalling is increased (Marfan's, Duchenne Muscular Dystrophy) show improved skeletal and cardiac muscle outcomes (Cohn et al., 2007). In humans, a controlled trial of losartan in renal transplant patients showed factors associated with tubular damage and graft fibrosis were reduced after eight weeks treatment with losartan as opposed to carvedilol, a β-blocker (Tylicki et al., 2007). Additionally, in a placebo-controlled trial of losartan in chronic allograft nephropathy lasting one year, 14 of 22 (63.6 %) treated patients showed stable or improved graft functions as opposed to 4 of 24 (16.7 %) in the placebo group. Due to the pathophysiological role that TGFβ appears to play in OI and the ability of Losartan to inhibit TGFβ-induced effects in other conditions, we hypothesised that losartan would reduce TGFβ signalling in cellular and mouse models of OI and that losartan would reduce bone resorption and increase bone mass providing a rationale for future use of this agent in OI patients.

## 2 Materials and methods

### 2.1 Cell culture

Immortalised human pre-osteoblasts (MG63) obtained from ATCC and primary human pre-osteoblasts (OB8A), a kind gift from Dr. Marianna Kruithof de Julio, University of Bern, were cultured in DMEM + Glutamax media supplemented with 10 % foetal calf serum (FCS). Healthy fibroblasts were isolated from frozen skin biopsies taken from patients with no gene mutations associated with OI (Balasubramanian et al., 2016). Patient fibroblasts were isolated from skin biopsies from two OI patients with COL1A1 mutations: a 9-year old male patient with a *COL1A1*, c.3421C>T in exon 47 pathogenic variant, resulting in truncated type 1 collagen; and a 4-year old male patient with a glycine substitution on *COL1A1* c599G > T, p,Gly200val in exon 8 (Balasubramanian et al., 2016). Four separate biopsies were taken from each patient. Osteogenesis Imperfecta phenotype-based assays on cultured fibroblasts and osteoblasts were carried out under IRAS Project ID: 220463; REC reference: (Ottewell et al., 2008)/YH/0026. Fibroblasts were cultured in fibroblast growth medium 3 (Sigma-Aldrich) supplemented with 10 % FCS, 1 ng/ml basic fibroblast growth factor and 5 μg/ml insulin. All cells were maintained at 37 °C in a humidified 5 % CO_2_ environment.

Cells were seeded at 2 × 10^4^ into 24-well culture plates. 72 h post seeding, 0 nM, 1 nM, 5 nM or 20 nM losartan (from a stock solution of 50 mg/500uL DMSO) was added and cells counted using a haemocytometer or collected and stored at −80 for RNA extraction.

### 2.2 In vivo murine studies

#### 2.2.1 OI mouse models

6-week old male OIM mice on a B6C3Fe a/a-Col1a2oim/J background were treated with 0 (control) 0.6 mg/L or 1.2 mg/L losartan via their drinking water for 28 days (*n* = 8 mice per group). 6-week old male OIM mice on a C57BL/6 background (Jax stock #001815) were treated with 0 or 0.6 mg/L losartan via their drinking water for 56 days (*n* = 7–8 mice per group). For gene expression studies 6–8 week old female C57BL/6 mice (obtained from Dr. Charlotte Phillips, University of Missouri) (*n* = 5/group) received 0 or 0.6 g/L losartan in their drinking water for 7-days or 40 μg TGFβ neutralising antibody (1D11.16.8; Bio X Cell) or 60 mg/kg SD108 as positive controls prior to the isolation of tissues for analysis. Serum was collected and stored at −80 °C for ELISA analysis, whole tibiae were isolated for down-stream analysis by μCT and histology whereas bone marrow was extracted from the femurs to analyse expression of genes associated with TGFβ signalling pathways and immune cells. Throughout the experiment, mice had free access to food and water and were maintained on a 12 h:12 h light/dark cycle. Experiments carried out using OIM mice were approved by the UConn Health Institutional Animal Care Committee (animal protocol number 101354-0419). All experiments utilising wildtype mice were carried out in accordance with local guidelines and with Home Office approval under project licence P99922A2E, University of Sheffield.

### 2.3 Microcomputed tomography imaging

μCT analysis was carried out using a Skyscan 1172 X-ray-computed μCT scanner (Skyscan, Aartselar, Belgium) equipped with an X-ray tube (voltage, 49 kV; current, 200 μA) and a 0.5-mm aluminium filter. Pixel size was set to 5.86 mm; for tibiae, scanning was initiated from the top of the proximal tibia as previously described (Ottewell et al., 2008).

### 2.4 Biochemical analysis

Serum concentration of collagen type 1 C-telopeptide (CTX) was measured using an autoanyliser (Cobas c701, c702, e411 and e602, Roche Diagnostics). TGFβ was measured using a commercially available TGFβ Quantikine ELISA kit in accordance with the manufacturer's instructions (DB 100C; R&D Systems, Abingdon, UK).

### 2.5 Histology

Tibiae were fixed in 4 % paraformaldehyde and analysed by μCT prior to decalcification in a solution of 1 % paraformaldehyde/0.5 % EDTA in PBS for 4 weeks, changing the solution at weekly intervals. Tibiae were embedded in paraffin, from which 4 μm sections were cut using a microtome. Osteoclasts were identified by tartrate-resistant acid phosphatase (TRAP) staining as previously published (Lefley et al., 2019) and osteoblasts were as mononuclear cuboidal cells residing as chains along the bone surface Osteoclasts and osteoblasts were quantified per millimetre of trabecular bone surface from whole sections using a Leica RMRB light microscope and OsteoMeasure Software™, as previously described (Parfitt et al., 1987).

### 2.6 Gene analysis

Total RNA was extracted using a RNeasy kit (Qiagen) and manufacturer's instructions before being reverse transcribed into cDNA using FastGene Scriptase II Ready Mix Kit (NIPPON Genetics). For pre-osteoblasts or OI fibroblasts equal quantities of cDNA from triplicates from each technical repeat were pooled prior to analysis of gene expression using real-time PCR and assays pre-loaded onto microfluidic array plates containing assays specific for OI, TGFβ and angiotensin signalling pathways and house-keeping genes (see supplementary Table 1): For effects on immune cells in the bone microenvironment cDNA from the bone marrow of 3 mice per group were analysed independently on microfluidic cards preloaded with assays designed to amplify the genes shown in supplementary Table 2. Each assay was loaded onto cards in duplicate and averaged before comparing expression with the housekeeping genes *GAPDH* and *18s*. Change in gene expression between normal and OI samples or treated and non-treated groups were assessed using ΔΔCT compared with appropriate control.

### 2.7 Protein analysis

OI fibroblasts, osteoblasts or femurs were snap frozen in liquid nitrogen following dissection and stored at −80 prior to protein extraction using the mammalian cell lysis kit (MCL-1, Sigma). 40 μg of protein was run on a bio-rad pre-cast 4–15 % gel then transferred onto PVDF membrane (Pierce, Thermofisher, UK). Nonspecific binding was blocked in 1× casein solution (Vector Laboratories) before incubation with primary antibody: Total SMAD2 rabbit monoclonal (#1302) at 1:150; phospho-SMAD2 rabbit monoclonal (138D4) at 1:200 (both from Cell Signaling) and GAPDH (ZG003) 1:200 (Thermofisher). HRP was detected using the Supersignal Chemiluminescence Detection Kit (Pierce). Band quantification was carried out using Quantity One Software (BioRad) and normalised to GAPDH and then unphosphorylated protein.

### 2.8 Statistical analysis

With the exception of gene expression data, analysis was performed using GraphPad Prism 9.1 (GraphPad Software Inc.) using ordinary one-way analysis (ANOVA) and Dunnett's post hoc test to determine statistical significance. Genes were considered to be significantly different for *P* values of <0.05 and an absolute fold change of >2. Gene ontology analysis was performed in R v4.2.1. Immune cell type signature scores were calculated as the geometric mean of the delta-Ct values for all genes in each cell-type signature. Signatures genes for each cell type were determined with reference to Nanostring document LBL-10043-08 (nCounter PanCancer Immune Profiling Panel Gene List.xlsx).

## 3 Results

### 3.1 Angiotensin and TGFβ signalling pathways are increased in fibroblasts isolated Type 1 OI patients compared with healthy controls

**Fig. 1:**
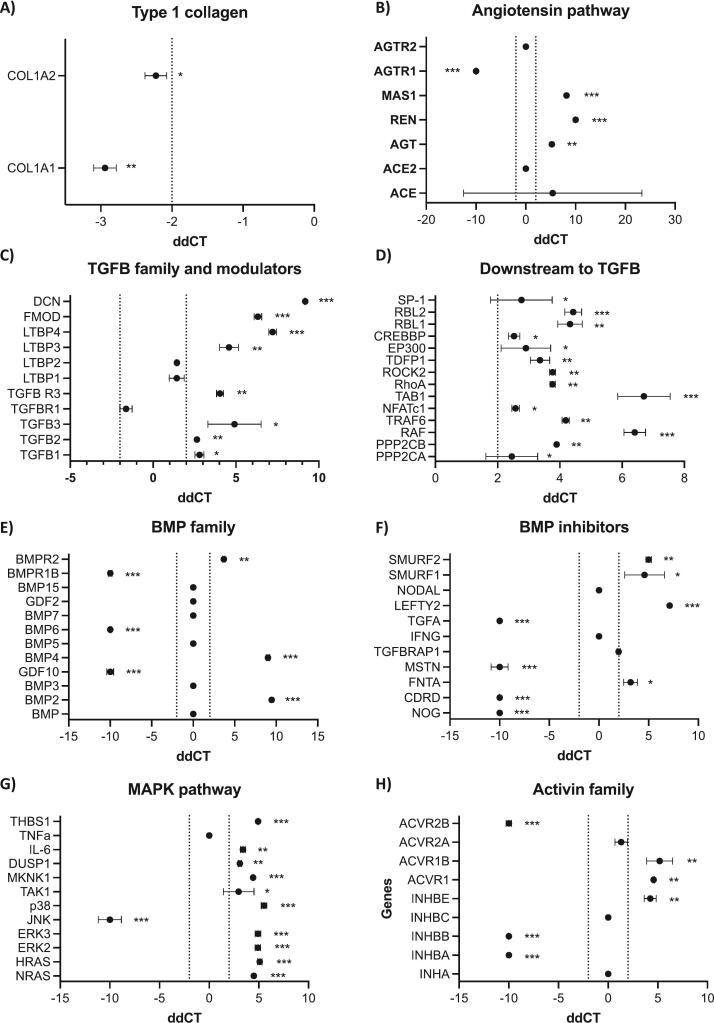
Comparison of angiotensin/TGFβ gene expression profiles between healthy and *COL1A1* mutated OI patient fibroblasts. Fibroblasts were isolated from normal (*n* = 2) and OI patients (n = 2) and cultured in triplicate, in vitro, for 6 days prior to analysis of gene expression by QPCR. Data shown are ddCT ± SEM with positive numbers representing an increase and negative numbers representing a decrease in gene expression in OI compared with normal fibroblasts for the type 1 collagen pathway (A), angiotensin pathway (B), TGFβ family and modulators (C), genes downstream of TGFβ (D), the BMP family (E), BMP inhibitors (F), the MAPK family (G) and the activin family (H). Data were considered significant if the change was >2 and *P* < 0.05. * = P < 0.05, ** = *P* < 0.01, *** = *P* < 0.001.

Type 1 OI is associated with pathogenic variants in *COL1A1* resulting in reduced type I collagen production. Patients with OI have increased TGFβ pathway activity in bone tissue (Bregou Bourgeois et al., 2016; Greene et al., 2021; Song et al., 2022). The angiotensin pathway has well documented effects on TGFβ activation and is known to contribute to osteoporosis (Mo et al., 2020). We hypothesised that targeting the angiotensin pathway with the angiotensin type 1 receptor blocker, losartan, may be a suitable option for treating OI. To provide mechanistic insight into the probability of losartan being effective as a treatment for OI we compared the gene expression profiles of the angiotensin and TGFβ signalling pathways in fibroblasts form Type 1 OI patients compared with healthy controls (Fig. 1).

QPCR analysis demonstrated a significant reduction in COL1A1 (*P* < 0.01) and COL1A2 (*P* < 0.05) in fibroblasts from Type 1 OI patients compared with healthy controls, providing confidence that our OI cells were reflecting gene expression profiles anticipated in OI (Fig. 1A). OI fibroblasts demonstrated significantly increased expression of genes associated with the angiotensin signalling pathway including AGT (*P* < 0.01), REN (*P* < 0.001) and MAS1 (P < 0.001) whereas the angiotensin receptor, AGTR1 was reduced (Fig. 1B). Genes associated with the TGFβ family and modulators (Fig. 1C) as well as genes downstream of TGFβ (Fig. 1D) and the MAPK pathway (Fig. 1H) were predominantly upregulated in OI fibroblasts compared with healthy controls. Importantly, genes within the activin signalling pathway ACVR1B (*P* < 0.01) and ACVR1 (P < 0.01) that have previously been shown to be associated with type V OI and that encode bone morphogenic protein type 1 of the TGFβ superfamily were also increased in OI fibroblasts compared with control. Changes in expression of BMP family members were variable, of note BMP4 (*P* < 0.001) and BMP2 (P < 0.001) were upregulated in OI compared with controls and the BMP inhibitors NOG (P < 0.001), CHRD (P < 0.001), MSTN (P < 0.001) and TGFA (P < 0.001) were downregulated (Fig. 1E and F). Taken together, these data suggest that targeting angiotensin signalling may be a suitable option for reducing TGFβ-induced bone pathology in OI.

### 3.2 Losartan reduces TGFβ secretion and normalises bone in OIM mice

**Fig. 2:**
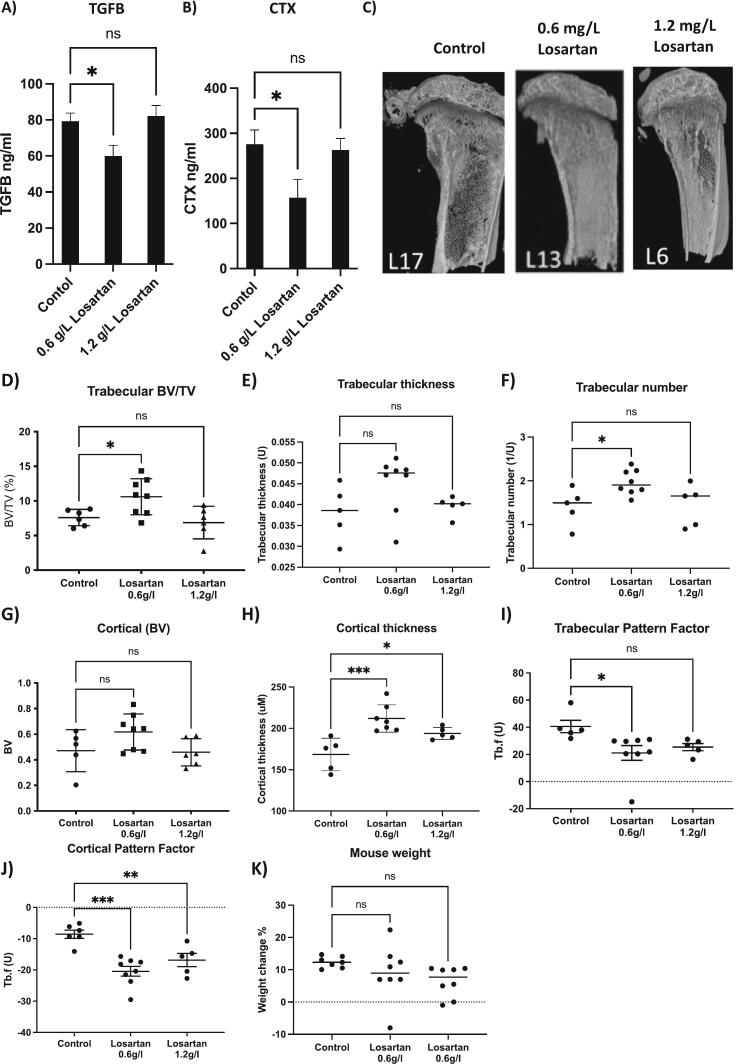
Effects of high and low dose losartan on TGFβ and bone parameters in OIM mice on a B6C3Fe a/a-Col1a2oim/J background. 6 week old male mice were treated with 0 (control) 0.6 mg/L or 1.2 mg/L losartan via their drinking water for 28 days (*n* = 8 mice per group for serum analysis; *n* = 5–8/group for analysis of non-fractured bone). A, shows mean ± SEM of TGFβ concentrations and B, shows CTX concentrations in mouse serum as measured by ELISA. C shows representative 3D μCT images of mouse tibiae. D shows % of trabecular bone volume to tissue volume (BV/TV%), E, trabecular thickness, F, trabecular number, G, cortical bone volume, H, cortical thickness, I and J trabecular and cortical pattern factor and K shows the percentage weight gain for mice over the 28-day experimental time period. Graphical data show mean ± SEM. * = P < 0.05, ** = P < 0.01, *** = P < 0.001.

To determine the potential efficacy of losartan in OI we first utilised an OIM mouse model on a mixed B6C3Fe a/a-Col1a2oim/J genetic background. Continuous administration of 0.6 g/L losartan via the drinking water for 28 days reduced serum TGFβ concentrations from 79.2 ± 14.6 ng·ml to 60.0 ± 18.6 ng/ml (*P* < 0.05) confirming that inhibiting the angiotensin receptor in OI mice effectively reduces TGFβ secretion (Fig. 2A). This treatment strategy resulted in serum concentrations of CTX being reduced from 275.9 ± 100.2 ng/ml to 157.2 ± 128.2 ng/ml (Fig. 2B), an increase in trabecular bone volume from 7.5 ± 1.1 % to 10.6 ±2.6 % (P < 0.05), a non-significant, increase in trabecular thickness (*P* = 0.061) and an increase in trabecular number (*P* < 0.05) (Fig. 2C–F). No significant alterations in cortical bone volume were observed, although there was a strong trend towards increased cortical bone volume following administration of 0.6 g/L losartan (*P* = 0.07) and a significant increase in cortical thickness (*P* < 0.001) (Fig. 2G–H). Interestingly, 1.2 g/L losartan had no effect on serum concentrations of TGFβ or CTX, nor did this dose of losartan affect trabecular bone volume (Fig. 2A–F). However, this higher concentration of losartan did increase cortical thickness (*P* < 0.05).

To assess bone quality trabecular and cortical pattern factors were measured; this parameter works on the understanding that a lot of concave structures indicate a badly connected pattern. Trabecular/cortical perforations lead to an increase in convex surfaces resulting in changes in pattern factor, with a reduction in pattern factor being associated with increased bone quality (Hahn et al., 1992). Administration of 0.6 g/L losartan resulted in reduced trabecular and cortical pattern factors (P < 0.05 and *P* < 0.001 respectively) whereas administration of 1.2 g/L losartan did not significantly reduce trabecular pattern factor but reduced cortical pattern factor (*P* = 0.07 and P < 0.05 respectively; Fig. 2I–J). Importantly this reduction in pattern factor was associated with reduced tibial fractures, with uCT revealing 38 % of tibiae were fractured in the control group, 0 % of tibiae fractured in mice receiving 0.6 g/L losartan and 25 % of tibiae fractured in mice receiving 1.2 g/L losartan. Fractures in other bones were not monitored; however the experiment was ended after 28 days of treatment due to control mice demonstrating limping behaviour. Because losartan was delivered in the drinking water, it is possible that reduced efficacy observed in mice administered 1.2 g/L compared with 0.6 g/L could be the result of mice drinking less water. Specific amounts of water consumed were not monitored, however, no visible differences were noted. As a surrogate measurement we assessed the weight gain in mice over the experimental time period (Fig. 2K). Data show no significant difference in weight between mice receiving losartan compared with control, however, there is a trend towards reduced weight gain in mice receiving 0.6 g/L (*P* = 0.46) and this trend increases in mice receiving 1.2 g/L losartan (*p* = 0.11). Taken together these data indicate that effects of losartan on TGFβ maybe dose dependent but it is more likely that mice receiving 1.2 g/L losartan received less drug due to consumption of less water.

**Fig. 3:**
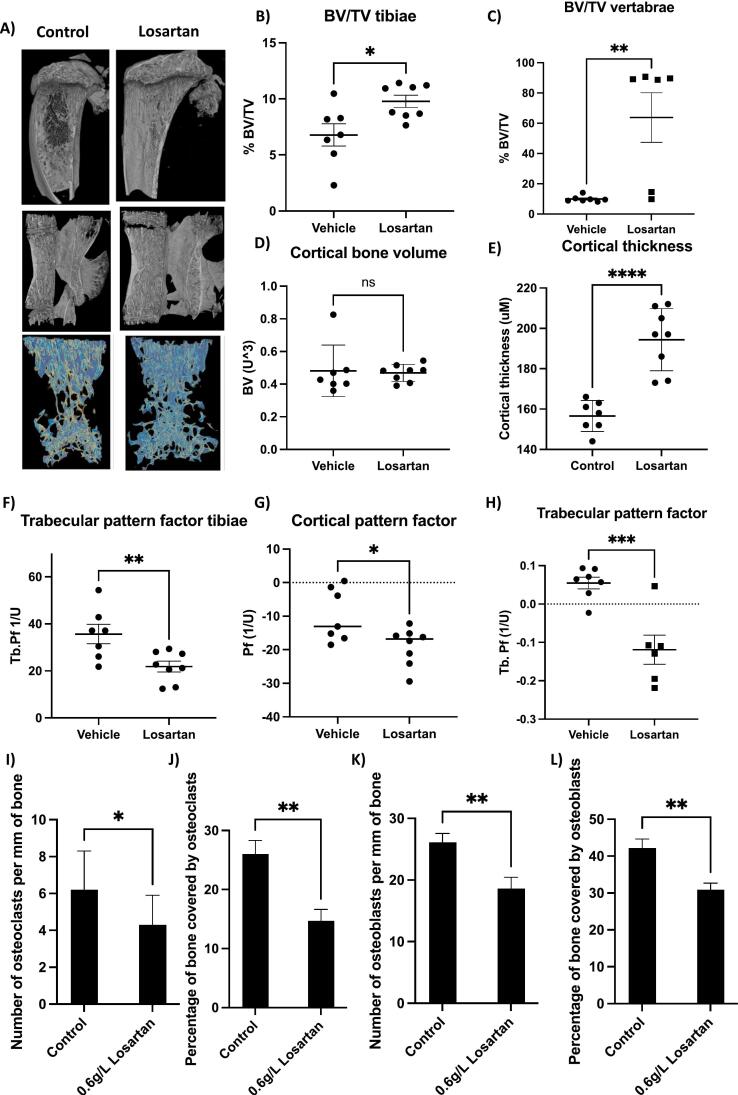
Effects of 0.6 mg/L losartan on bone parameters in OIM mice on a C57BL/6 background. 6-week old male mice were treated with 0 or 0.6 mg/L losartan via their drinking water for 56 days (*n* = 7–8 mice per group). A shows representative 3D μCT images of mouse tibiae. B and C show % of bone volume to tissue volume (BV/TV%) for the tibiae and vertebrae respectively. D and E show cortical bone volume and cortical thickness. F, G and H show pattern factor for trabecular bone in the tibiae, cortical bone in the tibiae and trabecular bone in the vertebrae respectively. Effects of losartan on osteoclast number and percentage of bone covered by osteoclasts are shown in I and J. Effects of losartan on numbers of osteoblasts and percentage of bone covered in osteoblasts are shown in K and L. All data are mean ± SEM. * = *P* < 0.05, ** = *P* < 0.01 and ***-*P* < 0.001.

**Fig. 4:**
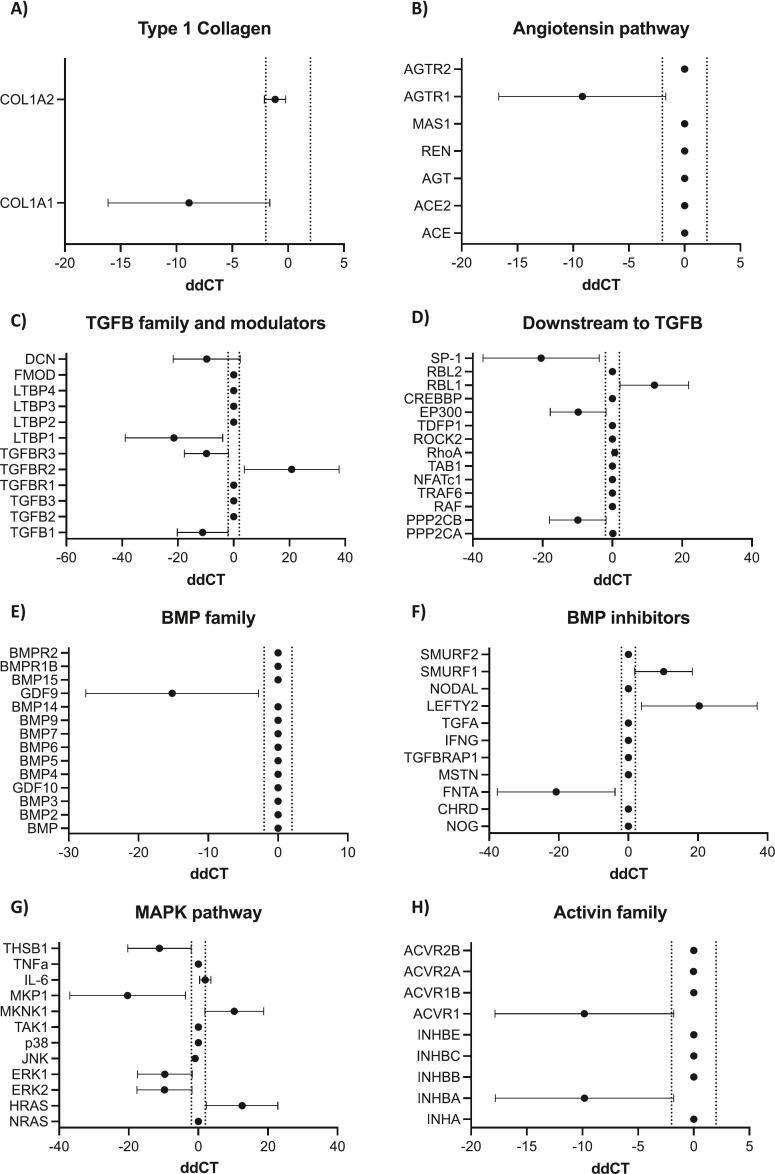
Effects of losartan on angiotensin and TGFβ gene expression pathways in *COL1A1* mutated OI fibroblasts. Fibroblasts were isolated from OI patients (*n* = 2), and cultured in triplicate, in vitro, for 72 h prior to administration of 0 or 5 nM losartan for a further 72 h. Samples were pooled for analysis (2 patients for sample) and analysed for gene expression by QPCR. Data shown are ddCT ± SEM with positive numbers representing an increase and negative numbers representing a decrease in gene expression in losartan treated compared with control OI fibroblasts for type 1 collagen (A), the angiotensin pathway (B), TGFB family and modulators (C), genes downstream of TGFB (D), the BMP family (E), BMP inhibitors (F), the MAPK pathway (G) and the activin family (H).

**Fig. 5:**
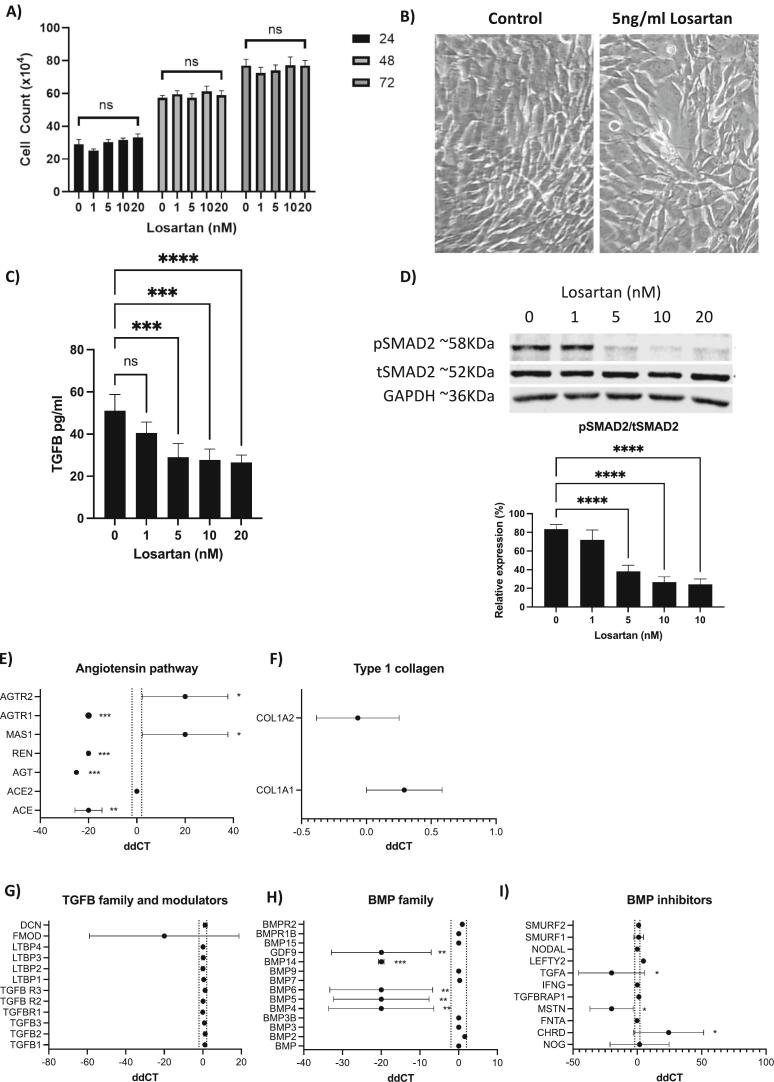
Effects of losartan on pre-osteoblast cells. Pre-osteoblast cells were treated with 0, 1, 5, 10 or 20 nM losartan for 24, 48 and 72 h. A shows mean ± SEM for numbers of pre-osteoblasts. B is a photomicrograph showing effects on pre-osteoblast differentiation. C shows mean ± SEM TGFβ concentrations in the culture media in pg/ml as measured by ELISA. D shows a representative Western blot for total SMAD2 (tSMAD2), phosphorylated SMAD2 (pSMAD2) and GAPDH protein taken from pre-osteoblasts treated with 0, 1, 5, 10 or 20uM losartan for 24 h, data quantification was from 3 independent experiments following normalisation to GAPDH and mean percentage of normalised phosphorylated protein to total protein ± SEM are shown in the histogram. E–I show ddCT ± SEM with positive numbers representing an increase and negative numbers representing a decrease in gene expression in losartan treated compared with control pre-osteoblasts. * = P < 0.05, ** = P < 0.01, *** = P < 0.001.

To validate findings from the OIM B6C3Fe a/a-Col1a2oim/J mouse model, effects of 0.6 mg/L losartan on bone parameters were assessed in a second OI mouse model which was bred on a pure C57BL/6 background (OIM C57BL/6 mice) (Fig. 3). This model displayed less severe bone pathology, allowing for effects of longer-term treatments to be assessed. Administration of 0.6 mg/L losartan in the drinking water of OIM C57BL/6 mice resulted in significant increases in trabecular bone volume compared with tissue volume in the tibiae (from 6.8 ± 2.6 % to 9.8 ± 1.5 %; *P* < 0.05) and vertebrae (from 9.6 ± 1.9 % to 63.77 ± 39.9; *P* > 0.01), furthermore, this dose of losartan resulted in increased cortical thickness (from 156 ± 7.3 to 194.4 ± 18.1; *P* < 0.001), (Fig. 3A–E) and caused a trend towards increased trabecular number and thickness (*P* = 0.64 and *P* = 0.51 respectively; supplementary Fig. 1) confirming that this dose of losartan is effective at increasing bone volume under OI conditions. Additionally, 0.6 mg/L losartan significantly reduced trabecular pattern factor in trabecular tibial (*P* < 0.01) and vertebral (P < 0.001) bone as well as cortical bone (*P* < 0.05); (Fig. 3D–H), suggesting that in addition to increased quantity of bone, administration of losartan also increased bone architectural quality. Histological analysis of tibiae from OIM C57BL/6 mice revealed that administration of 0.6 mg/L losartan led to significant reductions in numbers of osteoclasts (P < 0.05; Fig. 3I) and percentage of bone in contact with osteoclasts (P < 0.01; Fig. 3J) which may account for the increased bone volume observed following this treatment. Along with reduced osteoclasts, treatment with losartan also reduced numbers of osteoblasts (P < 0.01; Fig. 3K) and percentage of bone covered by osteoblasts (P < 0.01; Fig. 3L) indicating that reduced TGFβ signalling following losartan treatment leads to an overall reduction in bone turnover, resulting in increased bone volume and more regularly structured bone.

### 3.3 Effects of losartan on immune cell populations and TGFβ-associated gene expression in mouse bone

TGFβ plays prominent roles in immune cell regulation including stimulation of Treg cells and suppression of cytotoxic T cells (Jalalvand et al., 2021). As the bone marrow is the primary source of immature immune cells we assessed the potential impact of administration of losartan on immune cell populations to help predict any potential off-target effects that may be observed if this drug was administered as a long-term treatment for OI. We observed no significant differences in immune cell populations isolated from mouse bone marrow following administration of 0.6 mg/L losartan compared with control (supplementary Fig. 2) suggesting that this may be a safe treatment option.

To determine the mechanism by which losartan affects TGFβ signalling to increase bone volume and quality, gene expression profiles associated with angiotensin and TGFβ signalling pathways were analysed from cDNA extracted from control and losartan-treated whole mouse bone. For these experiments two additional control groups of mice were included, one in which mice were treated with an anti-TGFβ antibody and one in which mice were treated with a TGFBR1 antagonist (SD208) (supplementary Fig. 3). Gene expression data from whole mice bone demonstrated very few differences between control and losartan treated bone with significant reduction in gene expression only observed in ACVR1 and SMAD3 following losartan treatment (supplementary Fig. 3). Confirmation that losartan was effectively inhibiting TGFβ signalling was confirmed by reduced SMAD2 phosphorylation in the bones of mice treated with 0.6 g/L losartan compared with control (Supplementary Fig. 3). These data suggest that losartan-induced effects on TGFβ signalling may be cell type specific which is why we are not picking up specific gene expression changes in the whole bone cell population. We therefore performed further gene expression analysis on OI fibroblasts and osteoblasts to determine mechanism.

### 3.4 Losartan reduced TGFβ secretion and BMP gene expression in osteoblasts

Losartan has previously been shown to inhibit osteoclast number and differentiation through suppressing ERK 1/2 activation downstream of Ang II (Chen et al., 2015), however the effects of losartan on osteoblasts remain to be determined. To address this gap in our knowledge the effects of losartan on expression of genes associated with angiotensin and TGFβ signalling were assessed in healthy control or OI fibroblasts cultured, ex vivo, for 72 h before administration of 5 nM losartan for a further 72 h. Genetic analysis of cDNA isolated from these cells revealed no significant differences in expression of COL1A1, COL1A2 (Fig. 4A) or members of the angiotensin signalling pathway (Fig. 4B), and limited changes in members of the TGFβ signalling pathway (Fig. 4C) or genes known to be altered downstream of TGFβ (Fig. 4D) (data from OI fibroblasts shown in Fig. 5 and supplementary Fig. 3a; data from healthy controls, not shown). This lack of losartan-associated effects on the TGFβ signalling pathway in OI fibroblasts was confirmed by the failure to inhibit SMAD2 phosphorylation 72 h following exposure to 5uM losartan (supplementary Fig. 4b).

As we showed (see Fig. 3K–L) that losartan reduced osteoblast number in OIM mouse bone, the effects of losartan were subsequently assessed in pre-osteoblast cells, ex vivo. Administration of up to 20 nM losartan had no direct effect on proliferation of pre-osteoblasts (Fig. 5A), however, 5uM losartan for 72 h changed the morphology of cells increasing their capacity to differentiate (Fig. 5B). Unlike OI fibroblasts, which did not show altered TGFβ secretion following 72 h treatment with 5 nM losartan, this same treatment significantly reduced concentrations of TGFβ secreted into the culture medium by pre-osteoblast cells and reduced phosphorylation of SMAD2 (Fig. 5C and D). Gene expression analysis demonstrated significant reductions in AGTR1 (*P* < 0.001), REN (P < 0.001), AGT (P < 0.001) and ACE (*P* < 0.01) indicating significant reductions in the angiotensin pathway (Fig. 5E). Despite reductions in TGFβ secretion by pre-osteoblasts no significant alterations were observed in the expression of TGFβ associated genes and modulators (Fig. 5G), downstream modulators of TGFβ, the MAPK family or the SMAD family (supplementary Fig. 5). Importantly, expression of BMPs appeared to be significantly altered in pre-osteoblasts following losartan treatment GDF9 (P < 0.01), BMP4 (P < 0.01), BMP5 (P < 0.01), BMP6 (P < 0.01) and BMP14 (P < 0.001) were reduced in expression whereas the BMP inhibitor CHRD (*P* < 0.05) was increased and TGFA (P < 0.05) and MSTN (P < 0.05) were increased (Fig. 5H–I). It is therefore likely that reduced SMAD2 phosphorylation contributes to increased differentiation of pre-osteoblasts and improved bone morphology observed in OIM mouse models following losartan treatment.

## 4 Discussion

TGFβ, MAPK and activin pathways are commonly disrupted in OI (Etich et al., 2020; Shi et al., 2018) and high levels of TGFβ are hypothesised to play fundamental roles in driving the pathogenic mechanisms that are observed in OI bone (Grafe et al., 2014; Bianchi et al., 2015; Greene et al., 2021). In our current study we confirm upregulation of TGFβ and MAPK signalling pathways in primary fibroblasts isolated from OI patients with COL1A1 mutations, demonstrating that these cells retain their OI phenotype in culture. In addition to previously identified pathways in OI, increases in genes associated with the renin-angiotensin signalling pathway were also observed in OI fibroblasts compared with control. Although not previously described as being associated with OI, upregulation of the renin-angiotensin system is associated with increased collagen synthesis (Brilla et al., 1997), it is therefore possible that this pathway is upregulated as a compensatory response to reduced collagen synthesis in OI. Upregulation of angiotensin also leads to increased TGFβ synthesis, which could be responsible for the, as yet unexplained, increase in TGFβ observed in OI, however this hypothesis remains to be explored. Interestingly, we saw increased expression of Activin A receptor type 1 (ACVR1) and activin receptor 1B (ACVR1B) both of which play pivotal roles in TGFβ signalling with ACVR1 encoding bone morphogenic protein type 1 of the TGFβ superfamily and ACVR1 being involved in the recruitment of SMADs 2 and 3 (Hedger and de Kretser, 2013). Whilst a role for this gene in OI is uncertain, high expression may contribute to some bone effects in OI through the stimulation of TGFβ signalling pathways. Importantly, treating OIM/OIM or wild-type mice with soluble activin receptor has been shown to increase trabecular bone volume and improve cortical bone geometry and biochemical strength further suggesting that inhibiting effectors of TGFβ signalling may be a useful method of alleviating the adverse bone effects associated with OI without adversely affecting some related cell types (Jeong et al., 2018).

Similarly, to fibroblasts isolated from OI patients and other mouse models of OI (Greene et al., 2021; Song et al., 2022), OIM mice used in the current study (Phillips et al., 2000) expressed higher concentrations of plasma TGFβ (79.2 ± 14.6 ng/ml) compared with control, C57BL/6 mice (51.7 ± 6.1 ng/ml; data not shown). Administration of 0.6 g/L losartan but not 1.2 g/L losartan reduced TGFβ levels to those comparable to control (non-OI-C57BL/6 mice) suggesting that optimising dosing is critical for effective targeting of TGFβ signalling. Importantly, effects on bone resorption and bone mass were only observed following administration of losartan at concentrations shown to reduce TGFβ secretion (0.6 g/L) suggesting that reduced activity of TGF is critical for reduced bone resorption in this model of OI (Fig. 2). It must be noted, that in these experiments' losartan was delivered in the drinking water. This water was changed weekly and no visible differences in the amount consumed was observed, however, specific consumption was not measured. Mouse weights were used as a proxy for food/water consumption and, despite being in a better state of health, mice receiving losartan in their drinking water gained less weight (not significant) over the 28-day period of the experiment. It is therefore possible that mice receiving 1.2 g/L losartan drank less than those that received 0.6 g/L resulting in the reduced bone preserving effects observed with the higher dose of drug. Previously published data has suggested that direct targeting of TGFβ with the anti-mouse TGFβ antibody (1DII) also leads to reduced bone turnover and increased bone volume in the spine confirming our hypothesis that targeting this cytokine may be an effective treatment for OI (Greene et al., 2021). Interestingly, administration of 0.6 g/L losartan appeared to be at least as effective at reducing bone resorption compared with the previously published data using 1DII: CTX was reduced by over 40 % following losartan treatment and 25 % following 1DII treatment and bone volume in the spine was increased by 260 % following losartan and 235 % following 1DII. Whether differences in bone effects were due to the different OI mouse models or were treatment specific remain to be elucidated. Additionally, unlike targeting TGFβ, which has been shown to induce immunological events (Thomas et al., 2016) losartan did not alter immune cell populations (supplementary Fig. 3) in mice suggest that losartan may be an effective and safe treatment for increasing OI-associated low bone mass.

Analysis of mRNA from whole bone samples from mice treated with control or 0.6 mg/L losartan revealed no changes in the renin-angiotensin pathway and very few changes in the TGFβ signalling pathway which was unexpected given that TGFβ protein levels were significantly reduced and SMAD2 phosphorylation was inhibited. We therefore concluded that TGFβ and/or renin-angiotensin may only be significantly affected in a sub-set of cells in the bone environment and analysis of the whole bone may be diluting our gene expression data. In agreement with this idea, ex-vivo treatment of OI fibroblasts with losartan had no effect on SMAD2 phosphorylation nor did losartan affect genes associated with the renin-angiotensin pathway and only minimal effects on genes associated with the TGFβ family, their modulators, or genes directly downstream of TGFβ or the MAPK pathway. In contrast, losartan significantly reduced genes associated with the renin-angiotensin pathway (REN, AGT, ACE and AGTR1) as well as members of the BMP family (BMP4, BMP5, BMP6 and GDF9) in pre-osteoblast cells. Interestingly, losartan did not alter genes associated with the activin or the TGFβ family in pre-osteoblasts even though this treatment reduced TGFβ protein concentrations and SMAD2 phosphorylation (Fig. 5). Because angiotensin is known to stimulate canonical TGFβ through its type 1 receptor it is possible that reduced TGFβ seen following losartan treatment is the result of reduced expression of ANG and AGTR1 downregulating activation of this pathway (Ehanire et al., 2015). In the current study we used relatively low doses of losartan; 0.6 ml/L equates to 0.14 mg/kg and 1.2 mg/L equates to 0.28 mg/kg in mice that drink an average of 5.8mls water per day. For human patients, losartan is administered at a dose of 50-100 mg/day for high blood pressure and 12.5–150 mg for heart failure, equating to 0.58–1.17 mg/kg and 0.15–1.75 mg/kg in an average 85.4 kg man respectively, with a 100 mg dose resulting in peak plasma concentrations of 6.9uM 2 h after administration (Karra et al., 2012). Therefore, our data suggest that continuous administration of low dose losartan may be sufficient to reduce TGFB concentrations and improve bone health in patients with OI.

Osteoblast differentiation is a key step in skeletal development involving activation of several pathways including TGFβ and BMPs (Vimalraj and Selvamurugan, 2013) and this process is dysregulated in OI. In our ex vivo pre-osteoblast model, low doses of losartan 1-20 nM reduced the renin-angiotensin pathway and BMP-associated genes along with reducing TGFβ protein and SMAD2 phosphorylation. This treatment did not affect the ability of pre-osteoblasts to proliferate but simulated differentiation. Previously published data have demonstrated that 200 nM losartan also reduces osteoclast differentiation and activity (Chen et al., 2015) which may account for the reduced osteoclast numbers and activity observed in our OIM models following administration of losartan. Therefore, taking these data together as a whole, it is likely that reduced fractures, increased bone mass and reduced trabecular pattern factor (indicating normalisation of bone structure) seen in our OIM mouse models following treatment with losartan are the result of a combination of reduced osteoclastic bone resorption and increased osteoblast differentiation. It must be noted, however, that changes in osteoblast differentiation have only been observed morphologically and further studies are needed to determine the effects of losartan on osteoblast function. The effect of losartan on bone strength and long-term fracture risk in patients with OI remains to be elucidated, however, to date, our new data strongly suggest that losartan may be a suitable candidate for drug repurposing for the treatment of OI.

The following are the supplementary data related to this article.

Supplementary Table 1 Taqman gene expression assays to determine effects of losartan on TGFβ and angiotensin pathways. Supplementary Table 1

Supplementary Table 2 Taqman gene expression assays to determine effects of losartan on immune cell populations. Supplementary Table 2

Supplementary Fig. 1 Effects of 0.6 mg/L losartan on trabecular number and thickness in OIM mice on a C57BL/6 background. 6-week old male mice were treated with 0 or 0.6 mg/L losartan via their drinking water for 56 days (*n* = 7–8 mice per group). A, shows trabecular number and B, trabecular thickness as measured by uCT in the tibiae. Supplementary Fig. 1

Supplementary Fig. 2 Effects of losartan on immune cell populations in mouse bone. 6–8 week old female C57BL/6 mice were administered 0 or 0.6 μg/L losartan for 7 days (*n* = 5 mice per group). Bone marrow was isolated and gene expression assessed by QPCR. Data shown are ddCT gene expression profile representative of the individual cell types/immune checkpoints compared with 18s control. Supplementary Fig. 2

Supplementary Fig. 3 Effects of losartan on TGFβ signalling via SMAD inhibition. C57BL/6 mice were administered 0 or 0.6 μg/L losartan for 7 days (n = 5 mice per group). Bone marrow was isolated and gene expression assessed by QPCR. A shows a schematic of how losartan affects the TGFβ signalling pathway. B is a Western blot for total SMAD2 (tSMAD2), phosphorylated SMAD2 (pSMAD2) and GAPDH protein taken from mouse bone data quantification was from 3 independent experiments following normalisation to GAPDH and mean percentage of normalised phosphorylated protein ± SEM to total protein are shown in the histogram. C is ddCT gene expression profile compared with 0 μg/L losartan control and normalised to 18s control. * = *P* < 0.05, ** = *P* < 0.01, *** = *P* < 0.001. Supplementary Fig. 3

Supplementary Fig. 4 Effects of losartan on SMAD signalling gene expression in OI fibroblasts. OI fibroblasts were treated with 0 or 5 nM losartan for 72 h. Samples were pooled for analysis (2-patients for sample) and analysed for gene expression by QPCR or SMAD phosphorylation by Western blot. A shows ddCT ± SEM with positive numbers representing an increase and negative numbers representing a decrease in gene expression in losartan treated compared with control OI fibroblasts. B is a Western blot showing effects of 5 nM losartan on SMAD phosphorylation. Supplementary Fig. 4

Supplementary Fig. 5 Effects of losartan on MAPK, SMAD, activin, gene expression and pathways downstream of TGFβ. Pre-osteoblast cells were treated with 0, or 5 nM for 72 h. Data show ddCT ± SEM with positive numbers representing an increase and negative numbers representing a decrease in gene expression in losartan treated compared with control pre-osteoblasts. Supplementary Fig. 5

## CRediT authorship contribution statement

**Mai Morita:** Writing – review & editing, Investigation, Formal analysis, Data curation. **Fawaz Arshad:** Writing – review & editing, Investigation, Funding acquisition, Formal analysis, Data curation. **Lewis A. Quayle:** Writing – review & editing, Validation, Software, Methodology, Formal analysis. **Christopher N. George:** Writing – review & editing, Methodology, Investigation, Data curation. **Diane V. Lefley:** Writing – review & editing, Supervision, Investigation, Formal analysis, Data curation. **Ivo Kalajzic:** Writing – review & editing, Resources, Methodology. **Meena Balsubramanian:** Writing – review & editing, Resources, Methodology. **Tugba Cebe:** Writing – review & editing, Resources, Methodology. **Gwen Reilly:** Writing – review & editing, Resources, Methodology. **Nicolas J. Bishop:** Writing – review & editing, Supervision, Methodology, Funding acquisition, Conceptualization. **Penelope D. Ottewell:** Writing – original draft, Supervision, Resources, Project administration, Methodology, Funding acquisition, Conceptualization.

## Declaration of competing interest

NJB is global chief investigator of the Ultragenyx-funded studies (ORBIT, COSMIC) of setrusumab in children and young adults with OI and has consulted with Alexion, Mereo and Rampart and has been DMEC chair for a Pfizer study (recifercept in achondroplasia). No other authors have relevant conflicts of interest to declare.

## Data Availability

Data will be made available on request.
