# Supplementary material for: Losartan alters osteoblast differentiation and increases bone mass through inhibition of TGFB signalling in vitro and in an OIM mouse model
Source: Bone Rep. 2024 Jul 25;22:101795. doi: 10.1016/j.bonr.2024.101795 (PMC11344016; doi:10.1016/j.bonr.2024.101795)
Supplement: Supplementary Table 1 — Taqman gene expression assays to determine effects of losartan on TGFβ and angiotensin pathways. [file mmc1.pptx]

## Slide 1
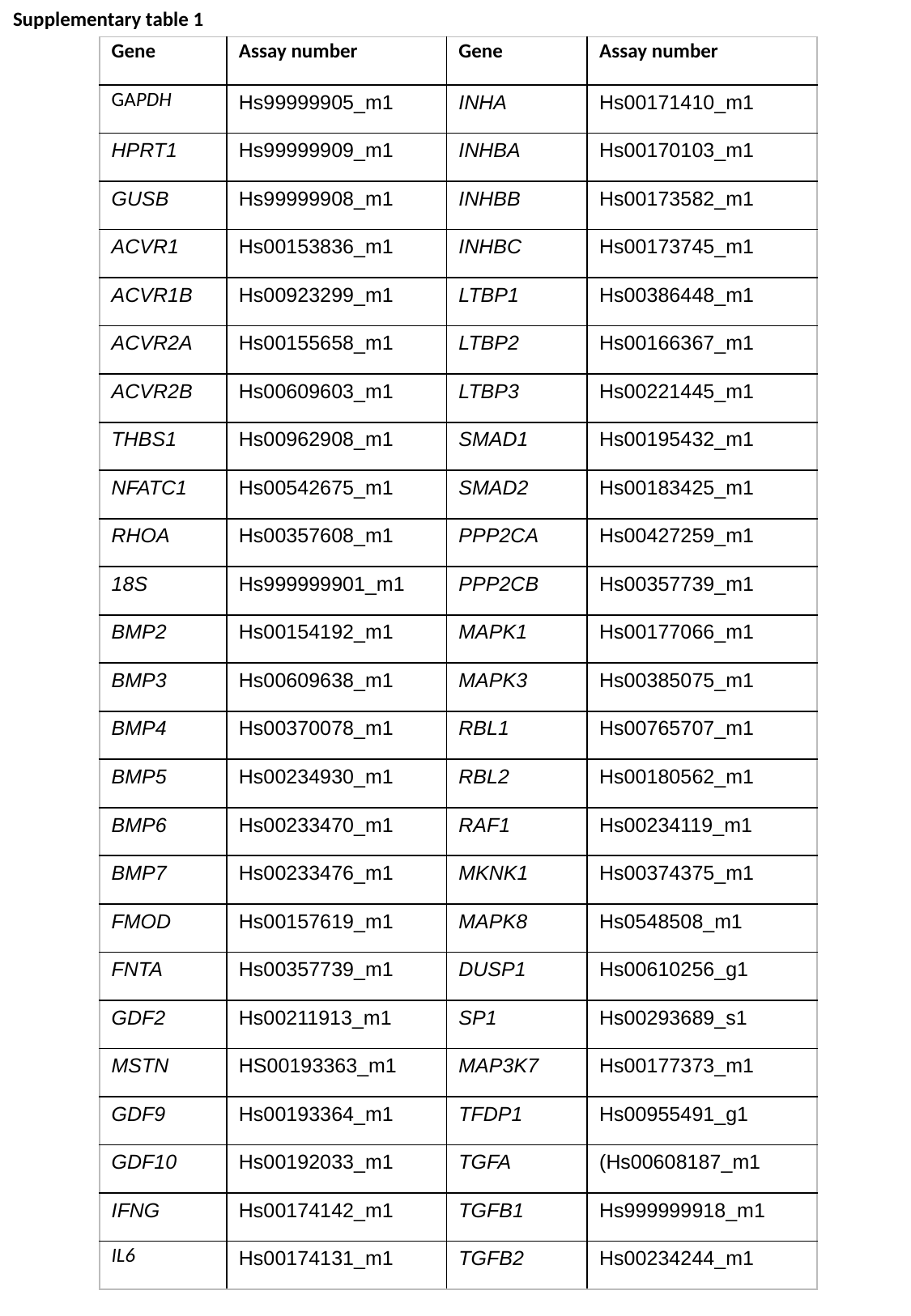

Supplementary table 1
| Gene | Assay number | Gene | Assay number |
| --- | --- | --- | --- |
| GAPDH | Hs99999905\_m1 | INHA | Hs00171410\_m1 |
| HPRT1 | Hs99999909\_m1 | INHBA | Hs00170103\_m1 |
| GUSB | Hs99999908\_m1 | INHBB | Hs00173582\_m1 |
| ACVR1 | Hs00153836\_m1 | INHBC | Hs00173745\_m1 |
| ACVR1B | Hs00923299\_m1 | LTBP1 | Hs00386448\_m1 |
| ACVR2A | Hs00155658\_m1 | LTBP2 | Hs00166367\_m1 |
| ACVR2B | Hs00609603\_m1 | LTBP3 | Hs00221445\_m1 |
| THBS1 | Hs00962908\_m1 | SMAD1 | Hs00195432\_m1 |
| NFATC1 | Hs00542675\_m1 | SMAD2 | Hs00183425\_m1 |
| RHOA | Hs00357608\_m1 | PPP2CA | Hs00427259\_m1 |
| 18S | Hs999999901\_m1 | PPP2CB | Hs00357739\_m1 |
| BMP2 | Hs00154192\_m1 | MAPK1 | Hs00177066\_m1 |
| BMP3 | Hs00609638\_m1 | MAPK3 | Hs00385075\_m1 |
| BMP4 | Hs00370078\_m1 | RBL1 | Hs00765707\_m1 |
| BMP5 | Hs00234930\_m1 | RBL2 | Hs00180562\_m1 |
| BMP6 | Hs00233470\_m1 | RAF1 | Hs00234119\_m1 |
| BMP7 | Hs00233476\_m1 | MKNK1 | Hs00374375\_m1 |
| FMOD | Hs00157619\_m1 | MAPK8 | Hs0548508\_m1 |
| FNTA | Hs00357739\_m1 | DUSP1 | Hs00610256\_g1 |
| GDF2 | Hs00211913\_m1 | SP1 | Hs00293689\_s1 |
| MSTN | HS00193363\_m1 | MAP3K7 | Hs00177373\_m1 |
| GDF9 | Hs00193364\_m1 | TFDP1 | Hs00955491\_g1 |
| GDF10 | Hs00192033\_m1 | TGFA | (Hs00608187\_m1 |
| IFNG | Hs00174142\_m1 | TGFΒ1 | Hs999999918\_m1 |
| IL6 | Hs00174131\_m1 | TGFΒ2 | Hs00234244\_m1 |

## Slide 2
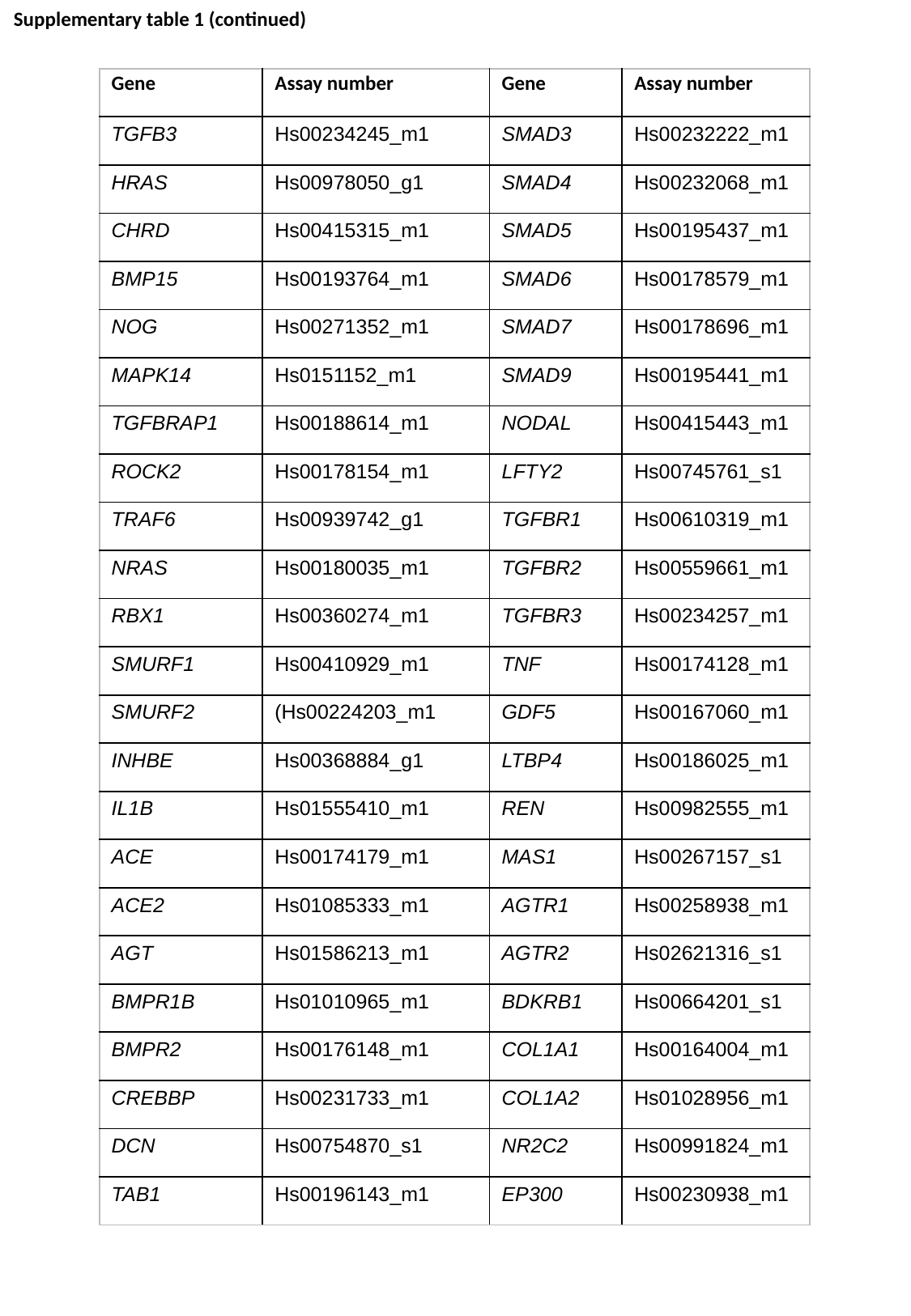

Supplementary table 1 (continued)
| Gene | Assay number | Gene | Assay number |
| --- | --- | --- | --- |
| TGFΒ3 | Hs00234245\_m1 | SMAD3 | Hs00232222\_m1 |
| HRAS | Hs00978050\_g1 | SMAD4 | Hs00232068\_m1 |
| CHRD | Hs00415315\_m1 | SMAD5 | Hs00195437\_m1 |
| BMP15 | Hs00193764\_m1 | SMAD6 | Hs00178579\_m1 |
| NOG | Hs00271352\_m1 | SMAD7 | Hs00178696\_m1 |
| MAPK14 | Hs0151152\_m1 | SMAD9 | Hs00195441\_m1 |
| TGFΒRAP1 | Hs00188614\_m1 | NODAL | Hs00415443\_m1 |
| ROCK2 | Hs00178154\_m1 | LFTY2 | Hs00745761\_s1 |
| TRAF6 | Hs00939742\_g1 | TGFΒR1 | Hs00610319\_m1 |
| NRAS | Hs00180035\_m1 | TGFΒR2 | Hs00559661\_m1 |
| RBX1 | Hs00360274\_m1 | TGFΒR3 | Hs00234257\_m1 |
| SMURF1 | Hs00410929\_m1 | TNF | Hs00174128\_m1 |
| SMURF2 | (Hs00224203\_m1 | GDF5 | Hs00167060\_m1 |
| INHBE | Hs00368884\_g1 | LTBP4 | Hs00186025\_m1 |
| IL1B | Hs01555410\_m1 | REN | Hs00982555\_m1 |
| ACE | Hs00174179\_m1 | MAS1 | Hs00267157\_s1 |
| ACE2 | Hs01085333\_m1 | AGTR1 | Hs00258938\_m1 |
| AGT | Hs01586213\_m1 | AGTR2 | Hs02621316\_s1 |
| BMPR1B | Hs01010965\_m1 | BDKRB1 | Hs00664201\_s1 |
| BMPR2 | Hs00176148\_m1 | COL1A1 | Hs00164004\_m1 |
| CREBBP | Hs00231733\_m1 | COL1A2 | Hs01028956\_m1 |
| DCN | Hs00754870\_s1 | NR2C2 | Hs00991824\_m1 |
| TAB1 | Hs00196143\_m1 | EP300 | Hs00230938\_m1 |
