# Supplementary material for: Losartan alters osteoblast differentiation and increases bone mass through inhibition of TGFB signalling in vitro and in an OIM mouse model
Source: Bone Rep. 2024 Jul 25;22:101795. doi: 10.1016/j.bonr.2024.101795 (PMC11344016; doi:10.1016/j.bonr.2024.101795)
Supplement: Supplementary Table 2 — Taqman gene expression assays to determine effects of losartan on immune cell populations. [file mmc2.pptx]

## Slide 1
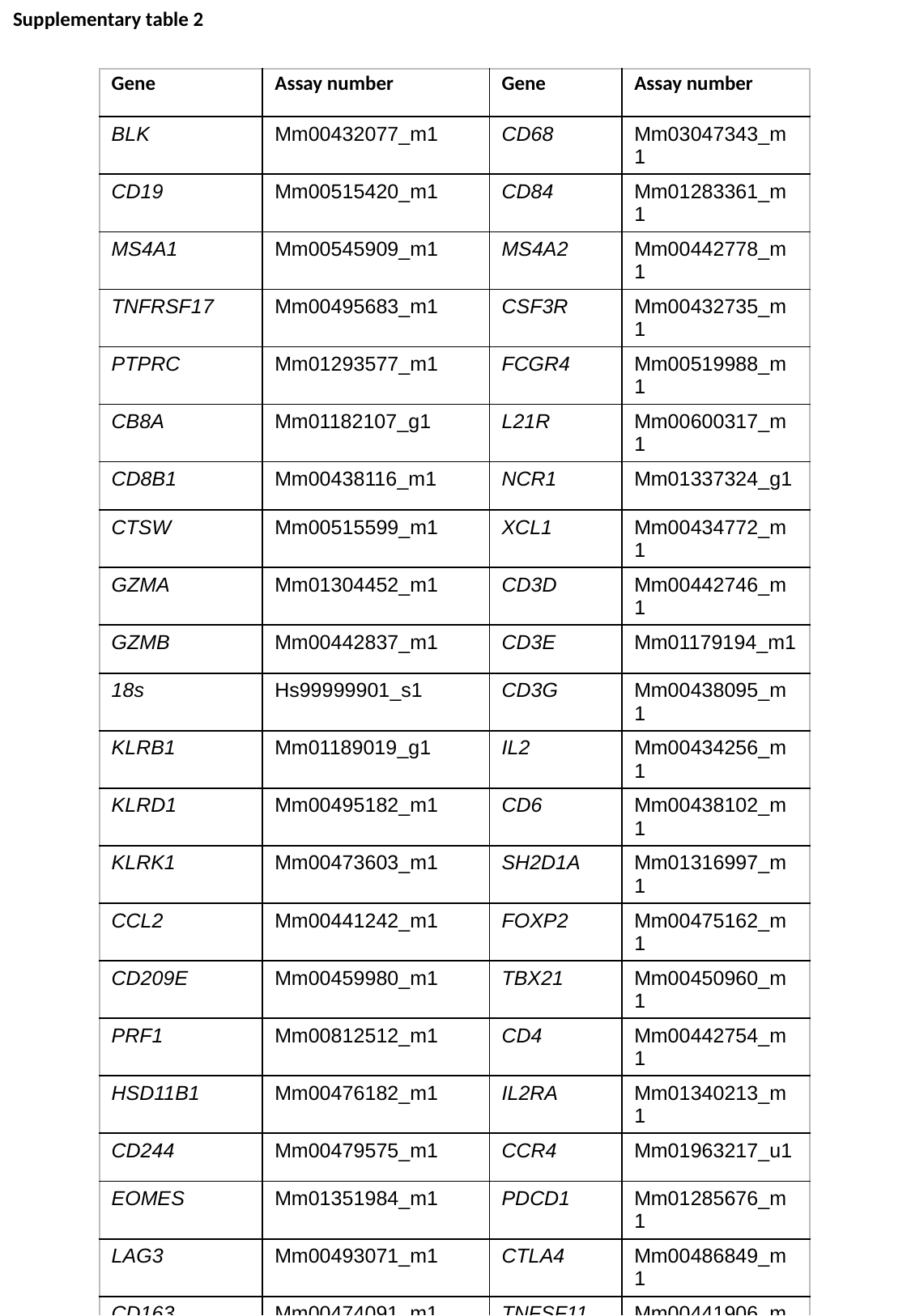

Supplementary table 2
| Gene | Assay number | Gene | Assay number |
| --- | --- | --- | --- |
| BLK | Mm00432077\_m1 | CD68 | Mm03047343\_m1 |
| CD19 | Mm00515420\_m1 | CD84 | Mm01283361\_m1 |
| MS4A1 | Mm00545909\_m1 | MS4A2 | Mm00442778\_m1 |
| TNFRSF17 | Mm00495683\_m1 | CSF3R | Mm00432735\_m1 |
| PTPRC | Mm01293577\_m1 | FCGR4 | Mm00519988\_m1 |
| CB8A | Mm01182107\_g1 | L21R | Mm00600317\_m1 |
| CD8B1 | Mm00438116\_m1 | NCR1 | Mm01337324\_g1 |
| CTSW | Mm00515599\_m1 | XCL1 | Mm00434772\_m1 |
| GZMA | Mm01304452\_m1 | CD3D | Mm00442746\_m1 |
| GZMB | Mm00442837\_m1 | CD3E | Mm01179194\_m1 |
| 18s | Hs99999901\_s1 | CD3G | Mm00438095\_m1 |
| KLRB1 | Mm01189019\_g1 | IL2 | Mm00434256\_m1 |
| KLRD1 | Mm00495182\_m1 | CD6 | Mm00438102\_m1 |
| KLRK1 | Mm00473603\_m1 | SH2D1A | Mm01316997\_m1 |
| CCL2 | Mm00441242\_m1 | FOXP2 | Mm00475162\_m1 |
| CD209E | Mm00459980\_m1 | TBX21 | Mm00450960\_m1 |
| PRF1 | Mm00812512\_m1 | CD4 | Mm00442754\_m1 |
| HSD11B1 | Mm00476182\_m1 | IL2RA | Mm01340213\_m1 |
| CD244 | Mm00479575\_m1 | CCR4 | Mm01963217\_u1 |
| EOMES | Mm01351984\_m1 | PDCD1 | Mm01285676\_m1 |
| LAG3 | Mm00493071\_m1 | CTLA4 | Mm00486849\_m1 |
| CD163 | Mm00474091\_m1 | TNFSF11 | Mm00441906\_m1 |
| NOS2 | Mm00440502\_m1 | CD274 | Mm03048247\_m1 |
