# Supplementary figures and images for: Losartan alters osteoblast differentiation and increases bone mass through inhibition of TGFB signalling in vitro and in an OIM mouse model
Source: Bone Rep. 2024 Jul 25;22:101795. doi: 10.1016/j.bonr.2024.101795 (PMC11344016; doi:10.1016/j.bonr.2024.101795)

Supplementary figure 1

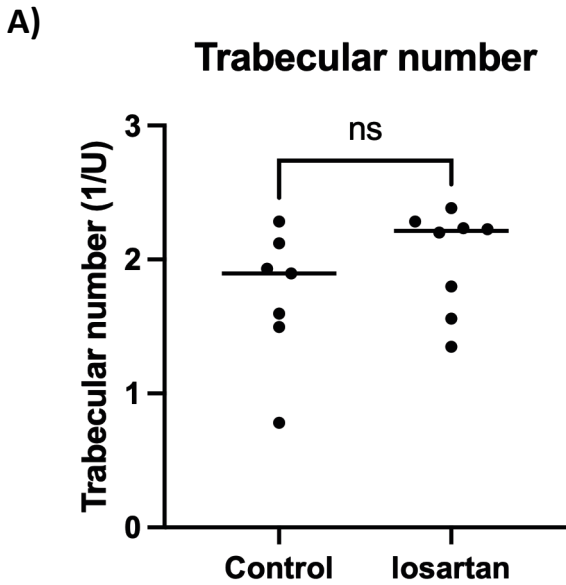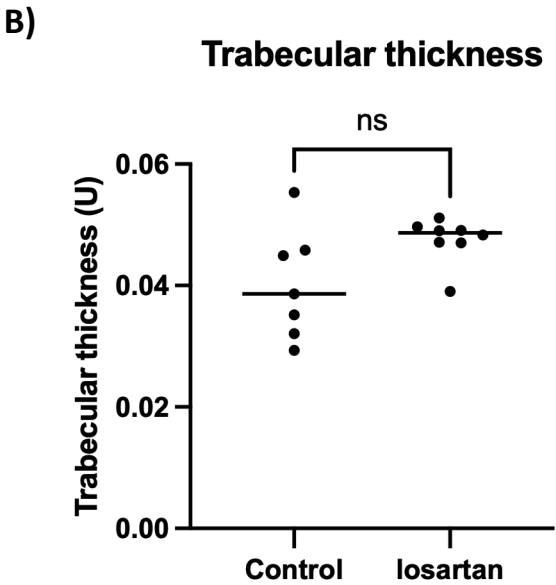

Supplement: Supplementary Fig. 1 — Effects of 0.6 mg/L losartan on trabecular number and thickness in OIM mice on a C57BL/6 background. 6-week old male mice were treated with 0 or 0.6 mg/L losartan via their drinking water for 56 days (n = 7–8 mice per group). A, shows trabecular number and B, trabecular thickness as measured by uCT in the tibiae. [file mmc3.pdf]

Supplementary figure 2.

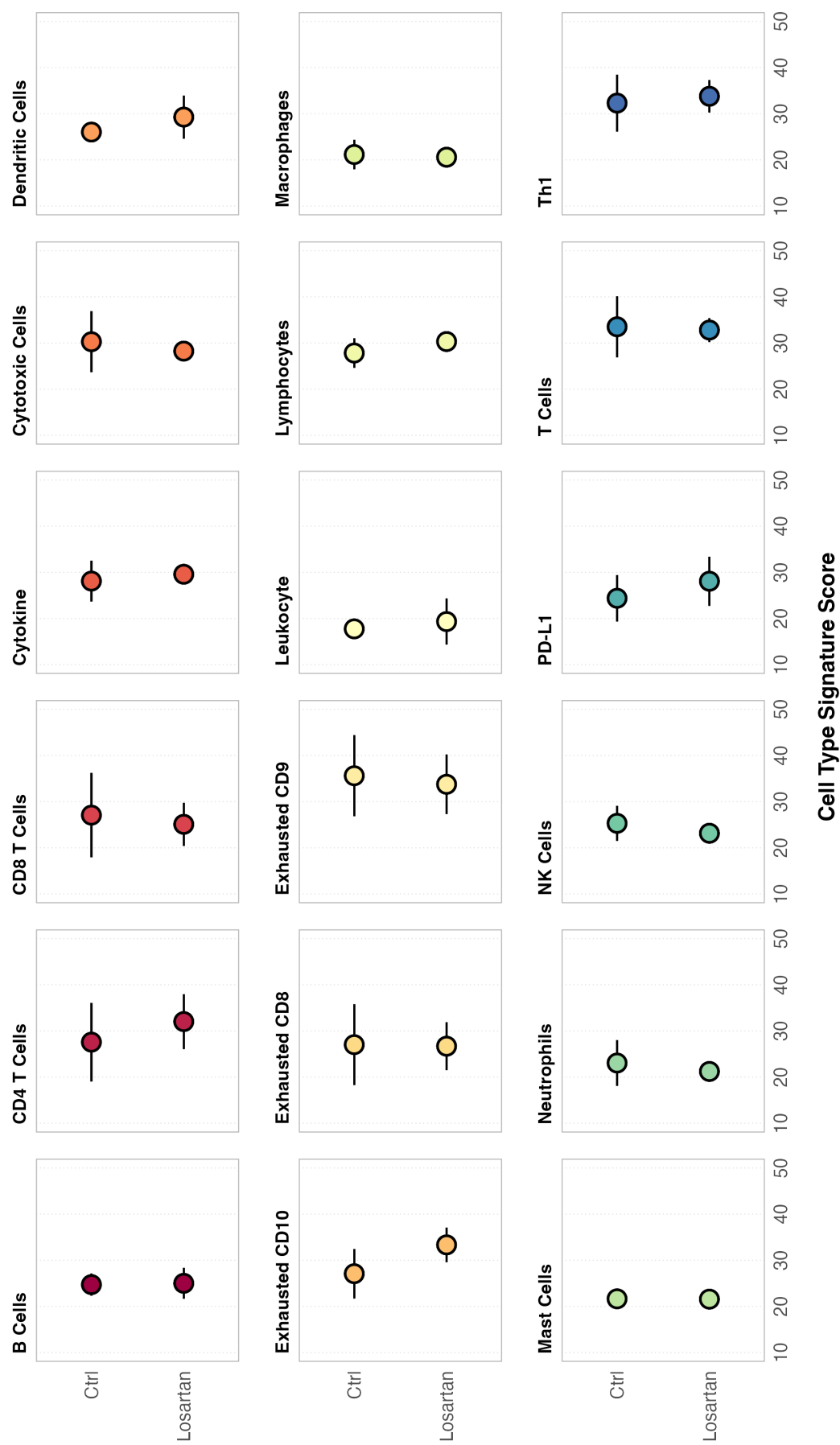

Supplement: Supplementary Fig. 2 — Effects of losartan on immune cell populations in mouse bone. 6–8 week old female C57BL/6 mice were administered 0 or 0.6 μg/L losartan for 7 days (n = 5 mice per group). Bone marrow was isolated and gene expression assessed by QPCR. Data shown are ddCT gene expression profile representative of the individual cell types/immune checkpoints compared with 18s control. [file mmc4.pdf]

Supplementary figure 3

A)

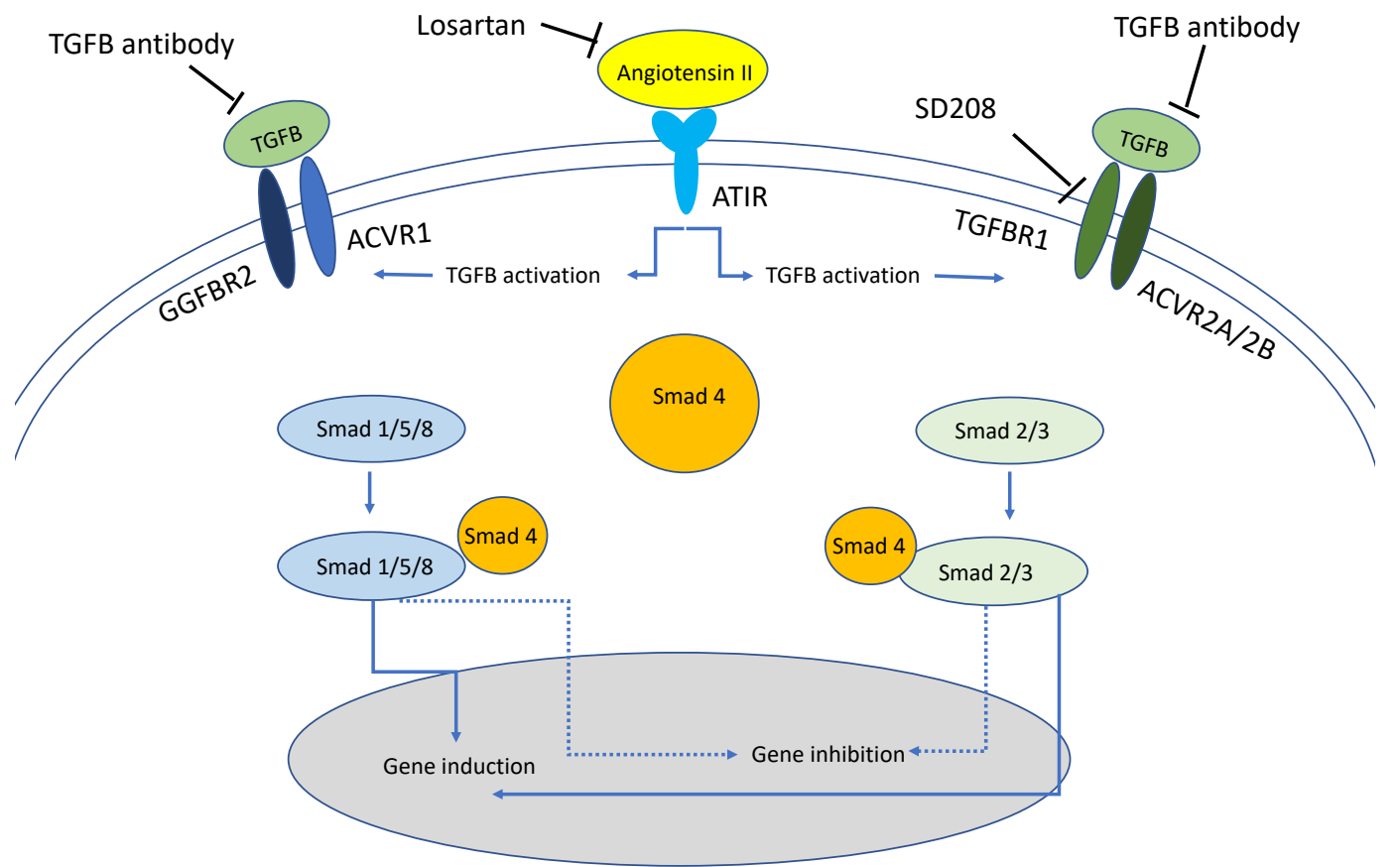

B)

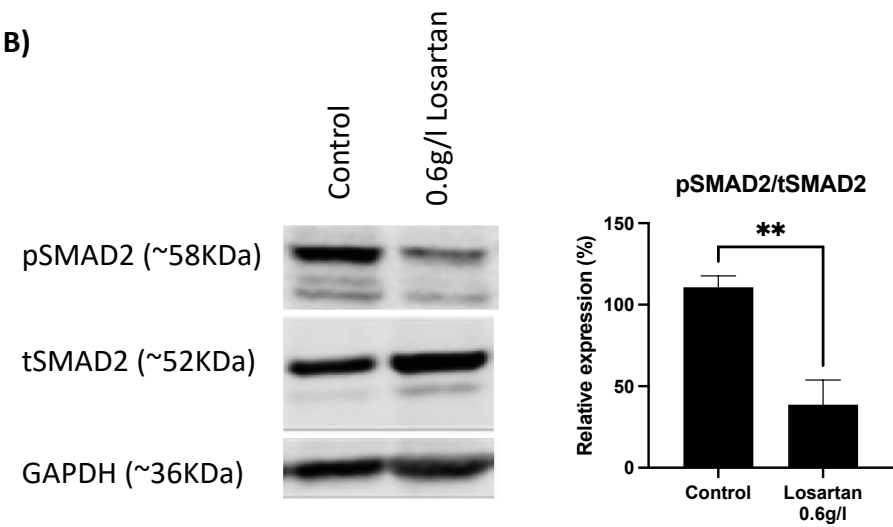

C)

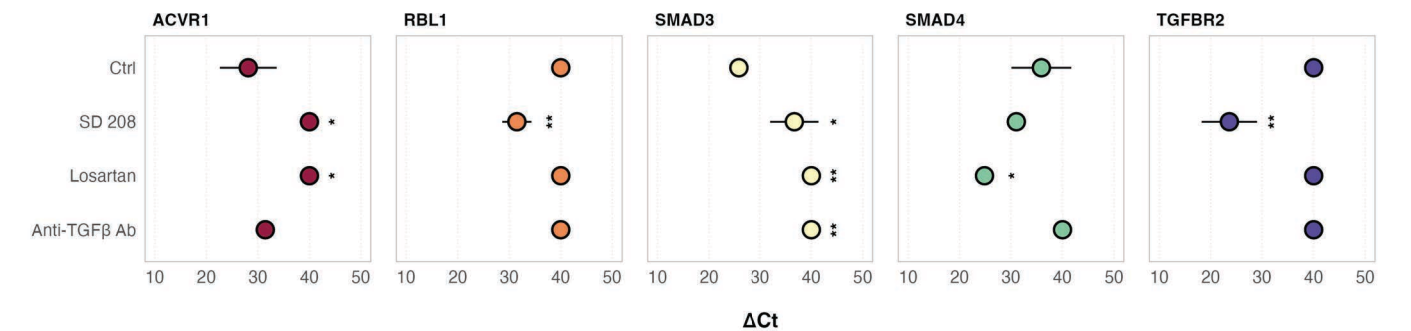

Supplement: Supplementary Fig. 3 — Effects of losartan on TGFβ signalling via SMAD inhibition. C57BL/6 mice were administered 0 or 0.6 μg/L losartan for 7 days (n = 5 mice per group). Bone marrow was isolated and gene expression assessed by QPCR. A shows a schematic of how losartan affects the TGFβ signalling pathway. B is a Western blot for total SMAD2 (tSMAD2), phosphorylated SMAD2 (pSMAD2) and GAPDH protein taken from mouse bone data quantification was from 3 independent experiments following normalisation to GAPDH and mean percentage of normalised phosphorylated protein ± SEM to total protein are shown in the histogram. C is ddCT gene expression profile compared with 0 μg/L losartan control and normalised to 18s control. * = P < 0.05, ** = P < 0.01, *** = P < 0.001. [file mmc5.pdf]

## Slide 1
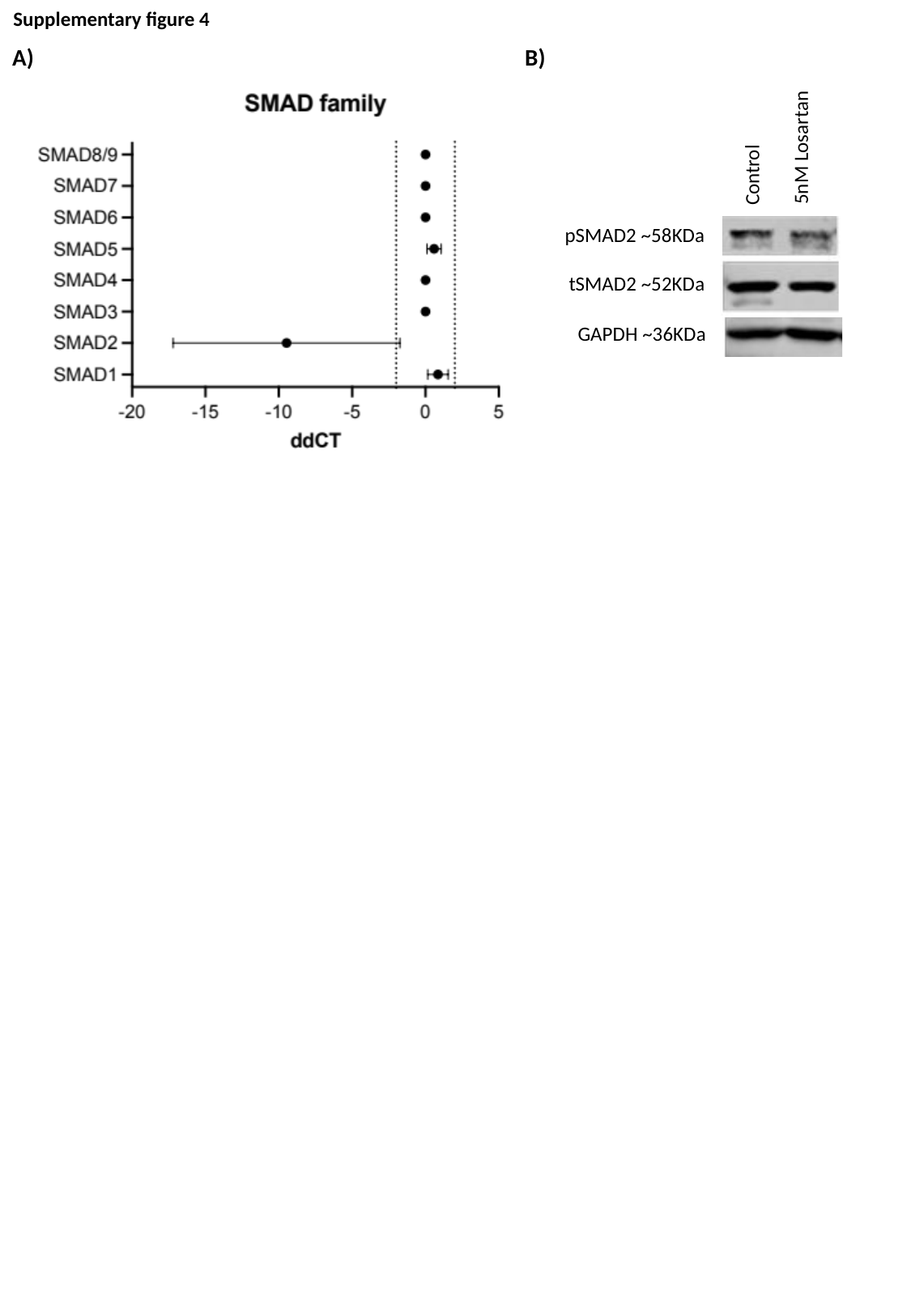

Supplementary figure 4
B)
A)
5nM Losartan
Control
pSMAD2 ~58KDa
tSMAD2 ~52KDa
GAPDH ~36KDa

Supplement: Supplementary Fig. 4 — Effects of losartan on SMAD signalling gene expression in OI fibroblasts. OI fibroblasts were treated with 0 or 5 nM losartan for 72 h. Samples were pooled for analysis (2-patients for sample) and analysed for gene expression by QPCR or SMAD phosphorylation by Western blot. A shows ddCT ± SEM with positive numbers representing an increase and negative numbers representing a decrease in gene expression in losartan treated compared with control OI fibroblasts. B is a Western blot showing effects of 5 nM losartan on SMAD phosphorylation. [file mmc6.pptx]

Supplementary figure 5

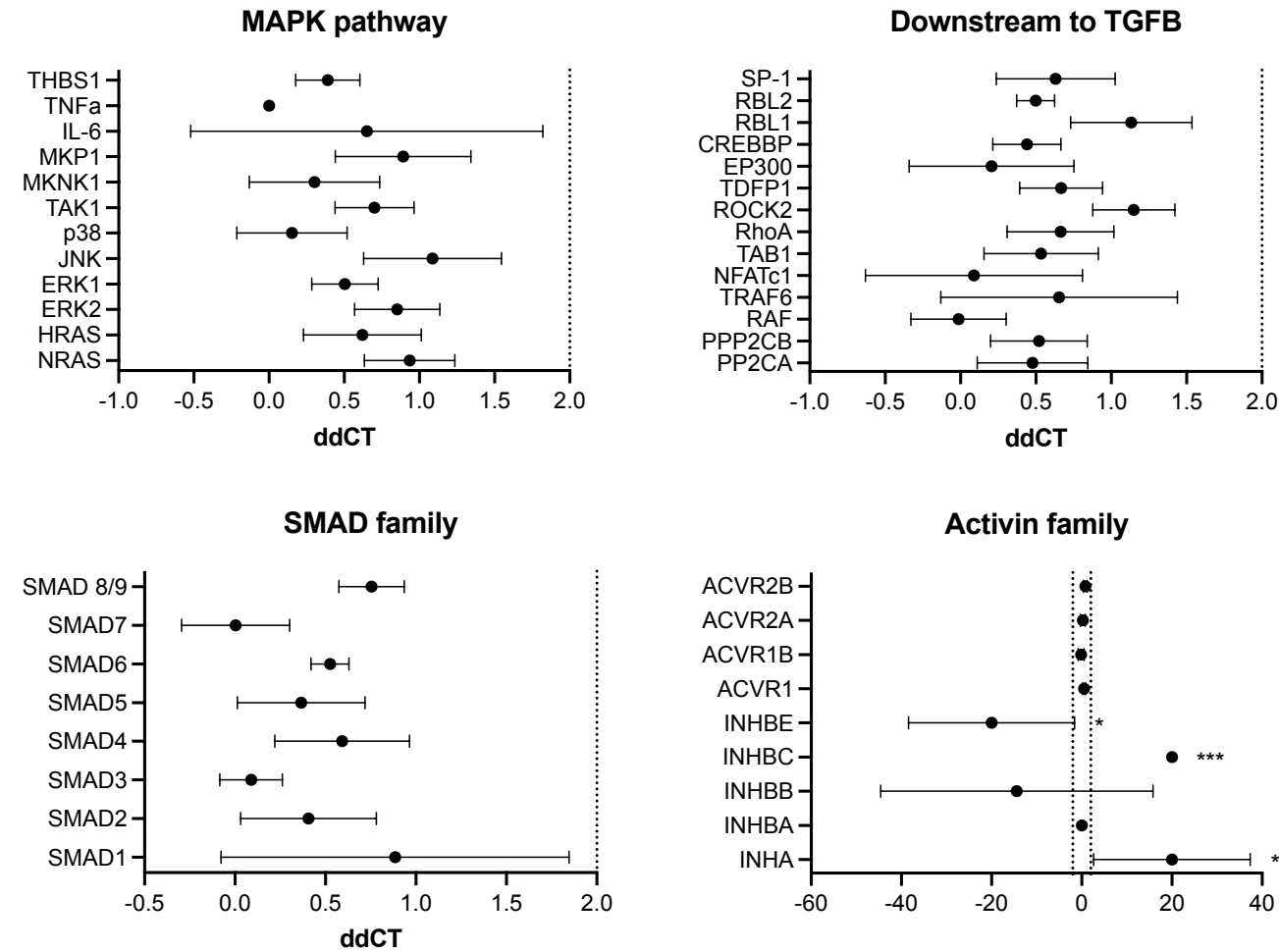

Supplement: Supplementary Fig. 5 — Effects of losartan on MAPK, SMAD, activin, gene expression and pathways downstream of TGFβ. Pre-osteoblast cells were treated with 0, or 5 nM for 72 h. Data show ddCT ± SEM with positive numbers representing an increase and negative numbers representing a decrease in gene expression in losartan treated compared with control pre-osteoblasts. [file mmc7.pdf]
